# Lipedema and obesity: A narrative review and treatment protocol

**DOI:** 10.1016/j.jpra.2026.01.004

**Published:** 2026-01-19

**Authors:** Sanskruti Rathod, Sjaak Pouwels, Jeremias Schmidt

**Affiliations:** a Dr. Panjabrao Alias Bhausaheb Deshmukh Memorial Medical College Amravati, Maharashtra, India; b Department of Surgery, University Hospital OWL of Bielefeld University - Campus Klinikum Lippe, Detmold, NRW, Germany; c Department of Intensive Care Medicine, Elisabeth-Tweesteden Hospital, Tilburg, the Netherlands; d Clinic for Plastic and Aesthetic Surgery, Helios Hospital Berlin-Buch, Berlin, Germany; e Department of Surgical Oncology, Transplant- and General Surgery, Gdansk Medical University, Gdansk, Poland

**Keywords:** Lipedema, Obesity, Lymphedema, Liposuction, Compression therapy, Multidisciplinary approach

## Abstract

**Background:**

Lipedema is a chronic condition characterized by abnormal fat accumulation, primarily in the lower extremities, affecting mostly women. Despite improvements in diagnosis and treatment, lipedema is often misdiagnosed as obesity or lymphedema. Patients with obesity and lipedema propose a distinct clinical challenge in treating both diseases. Improved recognition and understanding are necessary to enhance diagnosis and treatment outcomes.

**Purpose of this review:**

Lipedema is thought to be hormonally driven, often manifesting during puberty, pregnancy, or menopause. It presents as disproportionate fat accumulation in the lower body, often with microvascular changes. Misdiagnosis as obesity or lymphedema leads to ineffective treatments like weight loss programs and bariatric surgery. Effective management involves both conservative and surgical approaches, as well as a tailored strategy for patients with both lipedema and obesity. The focus of this review is to summarize the current literature addressing adequate treatment regimens for patients with both diseases and based on the literature we propose a treatment protocol.

**Conclusion:**

Patients with concurrent lipedema and obesity propose a distinct clinical challenge, in which early recognition can benefit adequate treatment. A combination of conservative measures and surgical options, particularly liposuction and/or bariatric and metabolic surgery, can be beneficial in treating patients with both diseases. However future research is needed to assess the effect of different treat regimens.

## Introduction

Lipedema, a chronic and progressive condition affecting subcutaneous adipose tissue, is characterized by symmetrical enlargement of the lower limbs and, in some cases, the upper limbs, sparing the feet and hands. It predominantly affects women and is often misdiagnosed or mistaken for obesity or lymphedema, leading to delayed or inappropriate treatment.^1^^,^^2^ Lipedema presents with disproportionate fat deposition, pain, tenderness, and easy bruising, contributing to impaired mobility and reduced quality of life.^3^^,^^4^ Despite its prevalence of 6–10% depending on the study by Hansdorfer-Korzon,^5^ awareness and understanding of lipedema remain limited, leading to underdiagnosis and suboptimal management.^5^ A genetic predisposition has been observed, with cases often reported in families, suggesting a possible hereditary component. Studies have indicated an autosomal dominant inheritance pattern in some patients, though the precise genetic and molecular basis of lipedema remains unclear.^6^ Due to the lack of genetic or biological markers, diagnosis relies heavily on clinical features. This underlines the importance of awareness among healthcare providers to correctly identify and differentiate lipedema from other conditions.^4^

The pathophysiology of lipedema is complex and multifactorial, with proposed mechanisms including adipocyte hypertrophy, inflammation, fibrosis, microangiopathy, and lymphatic dysfunction.^6^^,^^7^ Additionally, hormonal influences and genetic predisposition have been implicated, as lipedema often manifests during periods of hormonal change, such as puberty, pregnancy, and menopause.^8^^,^^9^ Recent research highlights endothelial cell alterations and impaired lymphatic function as contributing factors to the progression of lipedema.^10^ Obesity frequently coexists with lipedema, compounding the complexity of diagnosis and management. While obesity primarily results from an imbalance between caloric intake and expenditure, lipedema involves abnormal fat deposition that is resistant to diet and exercise.^7^^,^^11^ This distinction is crucial, as conventional weight management strategies often prove ineffective in addressing lipedema, necessitating targeted interventions.

Treatment protocols for lipedema aim to alleviate pain, reduce swelling, and improve mobility and quality of life. Conservative approaches include complete decongestive therapy, manual lymphatic drainage, compression therapy, and specialized exercise regimens.^12^^,^^13^ Emerging therapies such as ketogenic diets and low-carbohydrate, high-fat regimens have shown promise in reducing symptoms and improving quality of life in lipedema patients.^14^^,^^15^ In advanced stages, surgical interventions like tumescent liposuction or water jet-assisted liposuction may be necessary to address persistent adipose tissue and improve mobility.^16^ This narrative review explores the relationship between lipedema and obesity, providing insights into their shared characteristics, distinguishing features, and evidence-based treatment protocols aimed at optimizing outcomes for affected individuals.

## Materials and methods

Pubmed, Embase and the Cochrane Library were searched from the earliest date of each database until the May 5, 2025. All three authors screened the search results for relevant literature addressing different aspects of lipedema and obesity, specifically looking for literature that addressed treatment of patients with both obesity and lipedema.

### Epidemiology and pathophysiology of lipedema

#### Epidemiology

Lipedema is a chronic, progressive disorder characterized by abnormal subcutaneous adipose tissue deposition, primarily affecting the lower extremities and, in some cases, the arms. It predominantly affects women, with an estimated prevalence of 11–15%^1^^,^^3^ in adult females. Although lipedema is relatively common, it remains significantly underdiagnosed due to its resemblance to obesity and lymphedema.^17^^,^^18^ Men are rarely affected, with reported cases typically associated with hormonal imbalances, chronic liver disease, or Klinefelter syndrome.^17^^,^^18^

The onset of lipedema often coincides with hormonal changes, such as puberty, pregnancy, or menopause, suggesting a hormonal influence in disease progression.^12^ Genetic predisposition has been implicated in the pathogenesis of lipedema, with familial clustering reported in up to 60% of cases.^1^ A recent genome-wide association study identified potential loci linked to lipedema, further supporting its hereditary basis.^7^^,^^19^ However, despite advances in understanding the genetic underpinnings, no definitive causative mutations have been identified to date.^20^^,^^21^

Geographically, lipedema is more prevalent among Caucasian women, with fewer reports among Asian and African populations.^20^^,^^22^ The lack of ethnic diversity in reported cases may be attributed to underdiagnosis or limited awareness of the condition in non-European populations.^23^

#### Pathophysiology

Lipedema pathophysiology is complex and multifactorial, involving adipose tissue hypertrophy, microvascular dysfunction, lymphatic impairment, and chronic inflammation.^24^^,^^25^ The hallmark of lipedema is disproportionate fat accumulation in the extremities, sparing the feet and hands, which distinguishes it from obesity.^26^^,^^27^

##### Adipose tissue dysregulation

Adipose tissue in lipedema is characterized by hypertrophy and hyperplasia, with increased deposition of extracellular matrix (ECM) proteins and collagen.^28^ These structural changes contribute to tissue stiffness and impaired lymphatic drainage.^14^^,^^29^ Studies have shown that lipedema adipose tissue exhibits altered expression of genes involved in lipid metabolism, inflammation, and angiogenesis, further promoting adipose tissue expansion.^2^^,^^30^

##### Microvascular dysfunction

Microvascular dysfunction seems to play a critical role in the progression of lipedema. Increased capillary permeability and reduced capillary density have been observed in lipedema, leading to tissue hypoxia and interstitial fluid accumulation.^31^^,^^32^ The resulting edema and inflammatory response perpetuate the cycle of tissue damage and adipose tissue expansion.^10^

##### Lymphatic impairment

Although lipedema is distinct from lymphedema, lymphatic dysfunction is often observed in advanced stages of the disease. Lymphoscintigraphy studies have demonstrated delayed lymphatic transport and increased lymphatic vessel permeability in lipedema patients, suggesting compromised lymphatic function.^33^^,^^34^ This impairment may contribute to tissue inflammation, fibrosis, and progressive adipose tissue remodeling.

##### Chronic inflammation and immune dysregulation

Chronic low-grade inflammation is a hallmark of lipedema, with elevated levels of pro-inflammatory cytokines such as TNF-α, IL-6, and MCP-1 detected in lipedema adipose tissue.^6^^,^^35^ Macrophage infiltration and immune cell activation contribute to adipose tissue remodeling and fibrosis, perpetuating the disease process.^36^^,^^37^ Recent studies have also highlighted the role of oxidative stress in promoting adipose tissue dysfunction in lipedema.^38^^,^^39^

#### Challenges of lipedema and obesity

##### Diagnostic overlap and misclassification

Lipedema is often misdiagnosed as obesity, leading to inappropriate management strategies and delayed treatment. Both conditions present with increased adiposity, but lipedema exhibits distinct clinical features, including bilateral, symmetrical enlargement of the lower extremities, sparing the feet and hands.^40^^,^^41^ In contrast, obesity results in generalized fat accumulation without the characteristic sparing of the distal extremities.^15^

Failure to recognize these distinguishing features contributes to misclassification and delays in initiating appropriate treatment. Studies have shown that up to 50% of women with lipedema are initially misdiagnosed with obesity, resulting in frustration and emotional distress.^42^^,^^43^

##### Resistance to conventional weight management

A hallmark feature of lipedema is its resistance to caloric restriction and traditional weight-loss interventions.^43^ Unlike obesity, where adipose tissue responds to caloric deficits, lipedema adipose tissue seems to be resistant to diet and exercise.^44^ This resistance is attributed to the pathological alterations in adipose tissue metabolism, impaired microvascular function, and chronic inflammation.^45^ As a result, patients with lipedema often experience minimal improvement despite adherence to rigorous weight-loss programs, leading to feelings of frustration and hopelessness.^9^^,^^46^

##### Psychosocial and emotional impact

The psychological burden of lipedema is profound, with many patients experiencing anxiety, depression, and body image dissatisfaction.^47^^,^^48^ The chronic nature of the disease, coupled with repeated failures of conventional weight-loss strategies, contributes to emotional distress and diminished self-esteem.^49^^,^^50^ A survey of 200 women with lipedema revealed that 65% reported feelings of isolation, while 45% experienced moderate-to-severe depressive symptoms.^51^

##### Limited awareness and training among healthcare providers

Despite its high prevalence, lipedema remains under recognized by healthcare professionals. A lack of standardized diagnostic criteria and limited training in recognizing the characteristic features of lipedema contribute to the high rates of misdiagnosis.^52^^,^^53^ A recent survey of primary care physicians revealed that only 30% were familiar with lipedema, highlighting the need for improved education and training to enhance diagnostic accuracy.^4^^,^^54^

### Diagnostics

#### Clinical diagnosis

The diagnosis of lipedema is primarily clinical, based on a thorough history and physical examination. Characteristic features include symmetrical enlargement of the extremities, sparing the feet and hands, increased sensitivity to touch, and a tendency to bruise easily.^13^^,^^55^

#### Staging and classification

Lipedema has traditionally been classified into three clinical stages based on morphological changes in adipose tissue and skin involvement:

• Stage 1: Smooth skin surface with enlarged subcutaneous fat tissue.
• Stage 2: Uneven skin surface with palpable nodules and fibrotic changes.
• Stage 3: Large, deforming lobules of fibrotic adipose tissue with significant skin alterations.^56^^,^^57^

Additionally, lipedema is categorized into five types according to the anatomical distribution of fat accumulation:

• Type I: Pelvis, buttocks, and hips.
• Type II: Thighs and knees.
• Type III: Lower legs and ankles.
• Type IV: Arms.
• Type V: Isolated calves.^8^^,^^58^

However, this staging system is increasingly regarded as insufficient in clinical practice. Recent studies emphasize that pain is the hallmark symptom of lipedema, and importantly, there is no consistent correlation between the degree of pain and the clinical stage of the disease.^1^, ^2^, ^3^ This suggests that staging based solely on morphological presentation may not fully capture disease severity or patient experience.

Emerging evidence further underscores the multifactorial burden of lipedema, with pain intensity strongly impacting physical activity and psychological wellbeing, regardless of disease stage.^3^^,^^33^^,^^34^ Aitzetmüller-Klietz et al.^3^ demonstrated a vicious cycle where pain limits mobility, worsens mental health, and contributes to disease progression. Similarly, Dudek et al.^4^ and study by Poojari et al.^59^ highlighted the strong psychosocial distress and depression associated with lipedema pain, which does not align consistently with anatomical staging. This is one of the reasons why the recently changed German S2K guidelines for the treatment of lipedema do not use the traditional classification system anymore.^52^ They recommended to avoid staging-based therapeutic conclusions. Instead, clinicians should emphasize on pain, quality of life, psychological burden, and other patient-reported outcomes as the relevant metrics for disease assessment.^52^

Additionally, findings from recent data^60^ reveal significant impairment in quality of life, self-esteem, and daily functioning, even in women with early-stage lipedema. These insights support the urgent need to shift from a purely morphological classification toward a more symptom-oriented and patient-reported outcome-based approach to better reflect disease burden and guide management.

#### Imaging modalities

Imaging studies can aid in differentiating lipedema from other conditions such as lymphedema and obesity. The most commonly used modalities include ultrasound, magnetic resonance imaging (MRI), and lymphoscintigraphy.

##### Ultrasound findings

Ultrasound can identify hyperechoic subcutaneous tissue with thickened septae, indicative of fibrosis and adipose tissue hypertrophy.^61^^,^^62^ Doppler ultrasound may also reveal impaired venous return and increased capillary permeability in lipedema patients.^11^^,^^63^

##### Magnetic resonance imaging (MRI)

MRI provides detailed visualization of adipose tissue distribution and identifies characteristic changes, including nodular hypertrophy and fibrosis, which distinguish lipedema from other conditions.^64^^,^^65^ MRI findings in lipedema include increased signal intensity in subcutaneous fat and thickened fascial planes, reflecting the underlying inflammatory and fibrotic changes.^66^^,^^67^

##### Lymphoscintigraphy

Lymphoscintigraphy assesses lymphatic function and can differentiate lipedema from lymphedema. In lipedema, lymphoscintigraphy typically shows normal or mildly delayed lymphatic transport, whereas lymphedema is characterized by delayed or absent lymphatic flow.^42^^,^^68^^,^^69^

#### Differentiating lipedema from other disorders

Accurate differentiation of lipedema from other conditions is essential for appropriate management. Key distinguishing features include:

Lipedema vs. Obesity: Lipedema presents with symmetrical fat deposition sparing the feet and hands, while obesity involves generalized fat accumulation.^70^ It needs to be stated that lipedema is not caused by obesity, nor does it lead to obesity. Coincidental obesity is proportional and affects the trunk as well. As stated these diseases are considered as two separate entities, however can occur simultaneously.^52^

Lipedema vs. Lymphedema: Lymphedema typically presents with unilateral or asymmetric limb swelling, pitting edema, and dermal thickening, whereas lipedema exhibits symmetrical, non-pitting edema with easy bruising.^2^^,^^70^, ^71^, ^72^ Table 1 shows the distinct features of disease like lipedema, lipohypertrophy, obesity and lymphedema.

**Table 1:** Differentiation between lipedema, lipohypertrophy, lymphedema and obesity.

	Lipedema	Lipohypertrophy	Obesity	Lymphedema
Fat accumulation	+++	+++	+++	+
Disproportion of the extremities to the trunk	+++	+++	+	+
Edema	-	-	+	+++
Pressure pain	+++	-	-	-
Symmetry	+	+	+	-

## Treatment of lipedema

### Conservative management

#### Complete decongestive therapy (CDT)

CDT remains the cornerstone of conservative management for lipedema. It includes manual lymphatic drainage (MLD), compression therapy, exercise, and skin care to reduce swelling and improve lymphatic flow. Studies indicate that CDT reduces pain and leg volume in patients with lipedema, although the effects may be temporary without continued therapy.^12^^,^^13^^,^^42^^,^^44^

Manual lymphatic drainage (MLD): MLD facilitates the movement of lymph fluid and reduces interstitial fluid accumulation. Amato et al.^18^ demonstrated the benefits of MLD in managing lipedema-related swelling.

Compression therapy: Intermittent pneumatic compression (IPC) or multi-layered compression bandaging can significantly reduce limb circumference and pain.^12^^,^^46^ Atan and Bahar-Özdemir^12^ found that IPC was as effective as CDT in reducing pain and volume in severe lipedema.

#### Exercise therapy

Exercise improves mobility, reduces pain, and enhances quality of life in patients with lipedema. Low-impact exercises such as swimming, cycling, and walking are recommended to prevent further adipose tissue proliferation and improve venous and lymphatic circulation.^13^^,^^46^

Donahue et al.^13^ showed that multimodal physical therapy, including MLD and compression, improved mobility and reduced pain in women with early-stage lipedema.

Resistance training can also help improve muscle tone and reduce fat deposition in affected areas.^51^

#### Nutritional and dietary approaches

Dietary interventions, particularly low-carbohydrate, high fat (LCHF) or ketogenic diets, have demonstrated promising results in managing lipedema. These diets aim to reduce insulin resistance and inflammation, factors hypothesized to play a role in lipedema pathogenesis; however, direct evidence linking dietary changes to improvements in lipedema-specific outcomes remains limited and largely extrapolated from related metabolic conditions.^14^^,^^15^^,^^41^ Sørlie et al.^14^ and Jeziorek et al.^15^^,^^41^ found that ketogenic and LCHF diets led to significant reductions in pain, leg volume, and fat mass in patients with lipedema. The LIPODIET study showed that ketogenic diets improved quality of life and pain scores in patients with lipedema.^14^^,^^64^ However it should be noted that some guidelines do not recommended these diets based on inconsistent evidence.^31^^,^^52^

## Pharmacological management

### Anti-inflammatory agents

Given the inflammatory nature of lipedema, anti-inflammatory agents, such as flavonoids and antioxidants, have been explored for symptom relief.

Westcott and Rosen^73^ highlighted the role of anti-inflammatory agents in reducing fibrosis and adipocyte hypertrophy in lipedema.

Siems et al.^29^ demonstrated the anti-fibrosclerotic effects of shock wave therapy, which may alleviate pain and inflammation.

### Hormonal therapies

Since hormonal imbalances may exacerbate lipedema, hormonal therapies have been considered to modulate estrogen activity and mitigate disease progression. However, the evidence supporting the use of hormonal therapy in lipedema remains limited.^10^^,^^63^

## Surgical management

### Liposuction in lipedema

Liposuction can be an effective treatment for reducing the pathological fat deposits in lipedema, alleviating pain, and improving mobility and quality of life. Tumescent and water-jet-assisted liposuction (WAL) are the preferred techniques as they minimize trauma to the lymphatic vessels.^16^^,^^19^^,^^72^ On the other hand Power Assisted Liposuction (PAL) has also been recommended in the recently changed German S2K guideline for the treatment of lipedema, and is also reimbursed by the Healthcare system in Germany.^52^

Stutz and Krahl^16^ demonstrated that WAL effectively reduced lipedema volume while preserving lymphatic structures, with significant improvement in patient-reported outcomes. Wollina and Heinig^72^ conducted a study on 111 patients with lipedema undergoing microcannular liposuction, showing substantial pain reduction and volume reduction postoperatively.

### Safety and efficacy of liposuction

Liposuction in lipedema is generally safe when performed by experienced surgeons. However, patients with comorbidities such as von Willebrand disease require careful preoperative planning and hematological evaluation to prevent bleeding complications.^74^

Schmidt et al.^74^ reviewed the management of liposuction in lipedema patients with von Willebrand disease, recommending preoperative desmopressin and postoperative monitoring. Schmidt et al.^74^ also proposed a management algorithm for performing large-volume liposuction in lipedema patients with von Willebrand disease, emphasizing safety and postoperative care.

### Long-term outcomes of liposuction

Long-term studies have reported sustained improvements in pain, mobility, and quality of life following liposuction for lipedema. However, postoperative lymphatic function monitoring and adherence to compression therapy are essential to prevent recurrence.^16^^,^^72^

Goodliffe et al.^75^ emphasized the need for postoperative surveillance and patient education to maintain long-term benefits after liposuction.

Pouwels et al.^33^^,^^34^ highlighted cases where misdiagnosed lipedema led to weight regain post-bariatric surgery, underscoring the importance of accurate diagnosis before considering surgical interventions.

## Emerging and adjunctive therapies

### Shock-wave therapy

Shock wave therapy has shown promise in reducing fibrosis, pain, and tissue stiffness associated with lipedema. Siems et al.^29^ reported the anti-fibrosclerotic effects of shock wave therapy, suggesting its role as an adjunct to liposuction.

### Stem cell therapy and adipose tissue regeneration

Preliminary research suggests that autologous stem cell transplantation and adipose tissue regeneration may offer therapeutic potential in lipedema management.^26^^,^^53^

Ernst et al.^53^ explored the use of cultured adipocytes to identify diagnostic markers and potential regenerative therapies for lipedema.

### Platelet-rich plasma (PRP) and growth factors

PRP and growth factors are being investigated for their role in modulating inflammation and promoting tissue repair in lipedema.^30^

Scalise et al.^30^ highlighted the potential benefits of PRP in enhancing adipocyte metabolism and reducing inflammation.

## Postoperative care and long-term management

### Compression and lymphatic drainage

Postoperative care following liposuction in lipedema patients includes prolonged compression therapy and MLD to maintain reduced limb volume and to prevent recurrence.^44^^,^^52^

Faerber et al.^52^ provided updated guidelines emphasizing the importance of post-liposuction care to prevent fibrosis and recurrence.

### Psychosocial support and quality of life

Psychosocial support is critical in lipedema management as patients often experience body image distress, depression, and anxiety.^4^^,^^51^^,^^58^

Dudek et al.^4^ emphasized the need for psychological interventions and counseling to improve quality of life in lipedema patients.

Chachaj et al.^58^ reported higher levels of emotional distress and disability in lipedema patients compared to obese counterparts, underscoring the need for multidisciplinary care.

## Role of bariatric surgery in lipedema

Bariatric surgery is generally ineffective in reducing the pathological fat deposits associated with lipedema. This has also been stated in the German S2K guideline for the treatment of lipedema.^52^ However, it may be beneficial in managing comorbid obesity and improving overall metabolic health.^19^^,^^33^^,^^34^

Pouwels et al.^33^^,^^34^ described cases where lipedema was misdiagnosed as obesity, leading to suboptimal outcomes after bariatric surgery. Cornely et al.^57^ highlighted persistent lipedema pain in patients following bariatric surgery, emphasizing the need for accurate diagnosis before considering weight-loss procedures. Table 2 summarizes several treatment approaches of lipedema.

**Table 2:** Summary of lipedema treatment approaches.

Treatment modality	Key components	Evidence and key findings	References
1. Conservative management			
1.1 Complete decongestive therapy (CDT)	- Manual lymphatic drainage (MLD) - Compression therapy - Exercise - Skin care	- Reduces pain and leg volume temporarily - Requires long-term adherence for sustained results	^12^^,^^13^^,^^46^^,^^48^
Manual lymphatic drainage (MLD	Facilitates lymph movement and reduces interstitial fluid	Amato et al. showed significant reduction in swelling and pain	^18^
Compression therapy	Intermittent pneumatic compression (IPC or multi-layered bandaging	Atan and Bahar-Özdemir demonstrated comparable outcomes to CDT	^12^^,^^25^
1.2 Exercise therapy	Low-impact exercises (swimming, cycling, walking and resistance training	- Improves mobility and quality of life - Reduces pain and prevents adipose tissue proliferation	^13^^,^^50^^,^^55^
1.3 Nutritional and dietary approaches	Low-carbohydrate, high-fat (LCHF and ketogenic diets	- Reduces insulin resistance and inflammation - LIPODIET study showed significant pain and volume reduction	14, 15, 16^,^^31^^,^^66^
2. Pharmacological management			
2.1 Anti-inflammatory agents	Flavonoids (Diosmin and antioxidants	- Improves venous function and reduces edema - Westcott and Rosen emphasized their role in reducing fibrosis	^73^^,^^76^
2.2 Hormonal therapies	Modulate estrogen activity	Limited evidence in modifying disease progression	^10^^,^^65^
3. Surgical management			
3.1 Liposuction in lipedema	Tumescent and water-jet-assisted liposuction (WAL)	- Most effective in reducing pathological fat deposits - Stutz and Krahl demonstrated reduced volume and pain with WAL	^17^^,^^21^^,^^74^
3.2 Safety and efficacy of liposuction	Hematological evaluation in patients with bleeding disorders	- Schmidt et al. recommended preoperative desmopressin for von Willebrand disease	^74^
3.3 Long-term outcomes of liposuction	Postoperative compression therapy and MLD to prevent recurrence	- Long-term improvements in pain, mobility, and quality of life - Goodliffe et al. emphasized the importance of postoperative care	^74^
4. Emerging and adjunctive therapies			
4.1 Shock wave therapy	Anti-fibrosclerotic and pain-reducing effects	Siems et al. demonstrated its role in reducing fibrosis and inflammation	^32^
4.2 Stem cell therapy and adipose tissue regeneration	Autologous stem cell transplantation for adipose tissue regeneration	Ernst et al. explored its potential in modulating adipocyte activity	^28^^,^^57^
4.3 Platelet-rich plasma (PRP) and growth factors	Modulates inflammation and promotes tissue repair	Scalise et al. highlighted PRP benefits in adipocyte metabolism	^33^
5. Postoperative care and long-term management			
5.1 Compression and lymphatic drainage	Post-liposuction compression and MLD	Faerber et al. emphasized their role in preventing fibrosis and recurrence	^48^^,^^56^
5.2 Psychosocial support and quality of life	Counseling, psychological support, and body image interventions	- Dudek et al. emphasized the importance of psychological support - Chachaj et al. highlighted higher distress levels in lipedema patients	^4^^,^^55^^,^^62^
6. Role of bariatric surgery in lipedema	Management of comorbid obesity and metabolic health	- Ineffective for reducing pathological lipedema fat - Pouwels et al. reported suboptimal outcomes post-bariatric surgery	^21^^,^^36^^,^^37^^,^^61^

### Dilemmas in treatment of obesity and concomitant lipedema

The coexistence of obesity and lipedema poses a significant clinical challenge due to overlapping features, leading to frequent misdiagnosis and ineffective treatment. Obesity primarily results from excessive caloric intake and reduced physical activity, while lipedema is a genetically and hormonally driven condition characterized by disproportionate adipose tissue deposition, particularly in the lower extremities, that is resistant to diet and exercise.^35^^,^^42^ Westcott and Rosen^73^ highlighted the complex interaction between adipose tissue and the lymphatic system, suggesting that inflammatory pathways and lymphatic dysfunction contribute to the persistence of lipedema even after weight loss. Misdiagnosing lipedema as obesity often leads to inappropriate management strategies, including bariatric surgery, which may result in minimal improvement in lipedema-associated symptoms.^19^^,^^33^^,^^34^

Pouwels et al.^34^ illustrated the negative consequences of misdiagnosed lipedema in patients who underwent bariatric surgery, reporting persistent mobility issues and recurrent pain despite significant weight reduction. Furthermore, Fink et al.^47^ observed that patients with concurrent obesity and lipedema experienced only partial reduction in leg volume post-bariatric surgery, emphasizing the need for accurate diagnosis and tailored interventions. This diagnostic ambiguity often delays the implementation of appropriate therapeutic strategies, perpetuating patient frustration and poor clinical outcomes.

### Treatment strategies for patients with both lipedema and obesity

Optimal management of patients with both obesity and lipedema require an individualized, multidisciplinary approach that addresses the unique pathophysiology of each condition. Bariatric surgery may be indicated to manage obesity and its metabolic consequences but has limited efficacy in reducing the pathological fat deposits associated with lipedema. Bast et al.^19^ and Pouwels et al.^33^^,^^34^ reported that while bariatric surgery effectively reduces overall body weight, it fails to significantly alleviate lipedema-related pain and limb volume. Therefore, combining bariatric surgery with liposuction, specifically water-jet-assisted liposuction (WAL), may offer better outcomes for patients with both conditions. Stutz and Krahl^16^ demonstrated that WAL effectively removes lipedema fat while preserving lymphatic structures, thereby reducing pain and improving mobility.

Compression therapy and manual lymphatic drainage (MLD) remain essential in managing lipedema post-bariatric surgery to prevent fluid retention and fibrosis.^42^ Additionally, Torre et al.^63^ emphasized the role of anti-inflammatory and antioxidant therapies in reducing adipose inflammation and mitigating lipedema progression. Schmidt et al.^74^ highlighted the importance of hematological evaluation before large-volume liposuction in patients with lipedema and von Willebrand disease to ensure safer surgical outcomes.

In conclusion, a combination of targeted interventions, including bariatric surgery for obesity management and liposuction for lipedema, coupled with conservative therapies, provides a more holistic approach to improving quality of life and reducing complications in patients with concomitant obesity and lipedema.

### Proposed algorithm for treatment of lipedema and obesity

Here we propose a treatment algorithm based on the limited availability of studies assessing patients with obesity and lipedema. This algorithm could guide clinicians treating patients with both diagnoses. However, this algorithm still needs to be externally validated in larger studies.

Step 1: Clinical Assessment

Confirm diagnosis of lipedema based on clinical signs (painful, symmetrical fat accumulation on lower limbs, sparing of the feet, negative Stemmer sign).^13^^,^^17^^,^^18^^,^^24^

Measure:

• BMI
• Waist-to-height ratio (WHtR) = Waist circumference ÷ height

WHtR is a sensitive tool for central obesity and can indicate fat distribution mismatch.^59^

Step 2: Stratify by BMI
A. BMI ≥ 40 kg/m²

Primary intervention: Bariatric surgery based on obesity treatment guidelines.^60^ If severe disproportion (trunk-leg mismatch) and disabling lipedema symptoms: Consider staged liposuction before or after bariatric surgery.^13^^,^^17^^,^^24^

B. BMI 35–39.9 kg/m²

• Primary intervention: Bariatric surgery as per obesity guidelines.^60^ Liposuction may be added if disproportion and symptoms persist post-weight loss or if fat is resistant.^13^^,^^17^^,^^24^
• Liposuction may be preferred first if there is significant lower body adiposity.^13^^,^^17^^,^^24^^,^^60^
• Alternatively, in selected cases with a marked mismatch between the trunk and legs, liposuction may be performed prior to gastric bypass.
• The Waist-to-Height Ratio (WHtR) can serve as a supportive tool to assess central versus peripheral fat distribution and help guide treatment sequencing.^59^

C. BMI 30–34.9 kg/m²

Decision between bariatric surgery or liposuction depends on:

• Severity of symptoms
• Fat distribution pattern
• Patient preference and functionality
• Liposuction may be preferred first if there's significant lower body adiposity.^13^^,^^17^^,^^24^

D. BMI < 30 kg/m²

Liposuction is the treatment of choice for symptom relief after conservative treatment has failed.^13^^,^^17^^,^^24^ Conservative therapies should support maintenance.

Step 3: Waist-to-Height Ratio (WHtR) – Adjunct Tool

WHtR > 0.5 → Suggests central obesity (truncal).

WHtR ≤ 0.5 → Suggests peripheral adiposity (lipedema dominant).

Use this to guide whether liposuction may be prioritized before bariatric surgery, even if BMI is high.^59^

Step 4: Conservative Measures (all BMI categories)

Lifestyle management (e.g., anti-inflammatory and insulin-sensitizing diets).^59^ Compression therapy, manual lymphatic drainage, physical activity, and psychological support.^13^^,^^17^^,^^24^

Latest recommendations emphasize the importance of self-care and self-management strategies as foundational components of lipedema treatment.^13^^,^^17^^,^^24^^,^^59^

These include:

• Daily compression use
• Self-lymphatic massage techniques
• Nutritional self-regulation
• Goal setting and tracking progress
• Access to support groups or coaching.

### Questionnaires to identify lipedema

A simplified lipedema screening questionnaire was developed and tested on 109 female patients. It demonstrated high diagnostic accuracy, with a predictive model reaching up to 91.2% accuracy. The tool is quick, easy to use, and effective for identifying potential lipedema cases during clinical assessments.^17^

The study by Rapprich et al.^76^ evaluated 25 lipedema patients before and 6 months after liposuction. Results showed a 6.9% reduction in leg volume, significant pain relief (VAS score dropped from 7.2 to 2.1), and improved quality of life. Overall, liposuction significantly reduced lipedema symptoms. The questionnaire that is used in the study is mentioned below in the table.^76^

### Limitations and directions for future research

**Figure 1:**
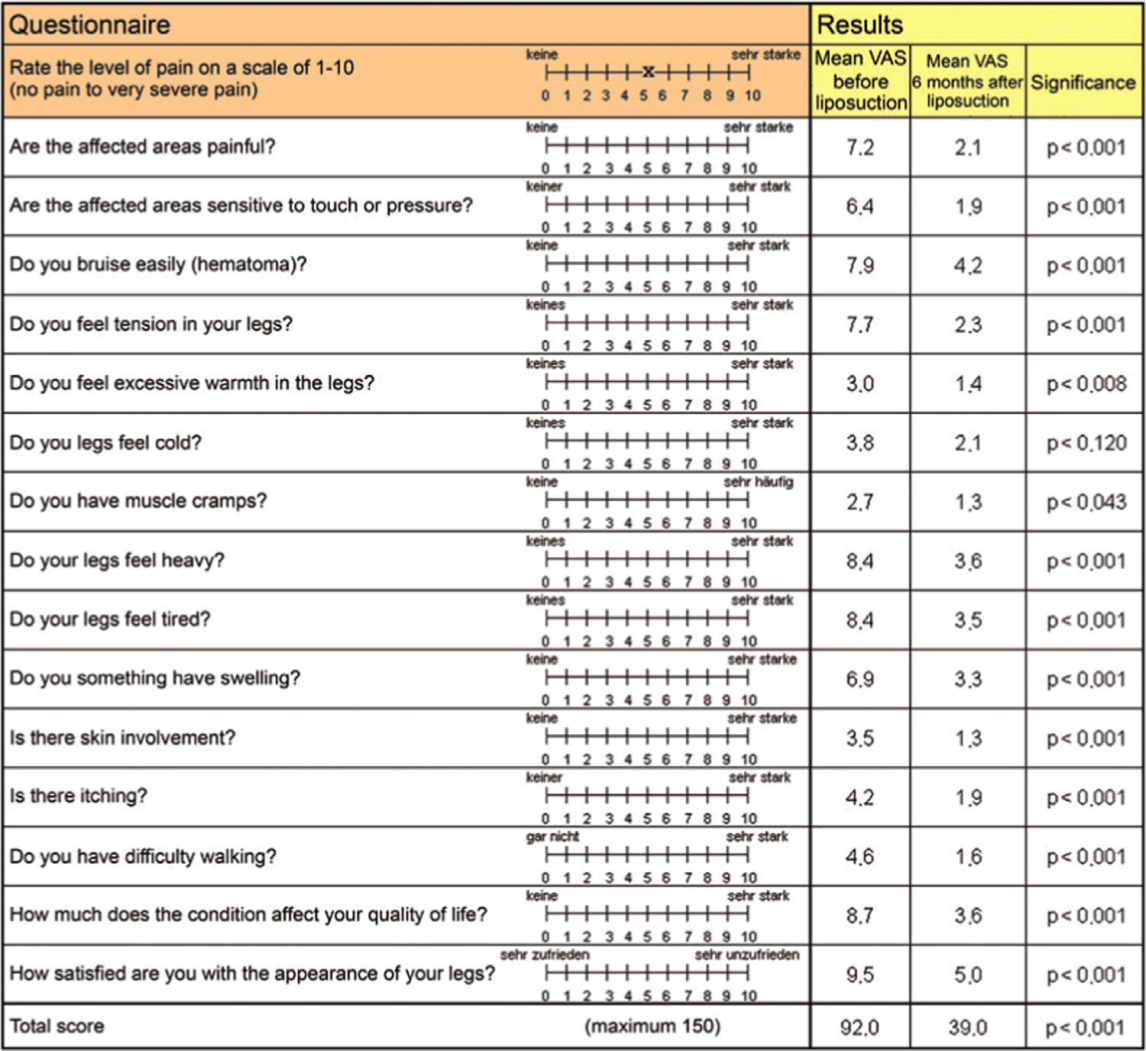
Questionnaire for measuring symptoms of lipedema by visual analogue scales. Adapted from Rapprich et al.^76^

Within this review we proposed a treatment algorithm based on currently available studies for patients with obesity and lipedema. This algorithm is based on a limited number of studies, but it addresses the need for such a treatment algorithm, since patients with both diagnoses can be very challenging to treat. This was substantiated by the studies done by Pouwels et al.^33^^,^^34^ and Cornely et al.^57^ This underscores an important fact in lipedema research. As stated before its prevalence is estimated around 11% in women worldwide, however it is unknown how many women with obesity also have lipedema. Also in the higher BMI’s, lipedema is very difficult to diagnose. Future research should focus on identifying the incidence and prevalence of lipedema in patients with obesity. Also its treatment post-bariatric surgery is a point of discussion. Based on the current literature, there is not much known when and how we should treat patients that had bariatric surgery and get a first diagnosis of lipedema post-bariatric surgery. Finally, for future research it should be investigated who should diagnose lipedema in patients with obesity. Since lipedema is a clinical diagnosis, experienced and/or well-trained physicians should diagnose and treat these patients (Figure 1).

## Conclusion

Patients with concurrent lipedema and obesity present a distinct clinical challenge, in which early recognition can benefit, adequate treatment. A combination of conservative measures and surgical options, particularly liposuction and / or bariatric and metabolic surgery, can be beneficial in treating patients with both diseases. However future research is needed to assess the effect of different treat regimens.

## Declaration of competing interest

None declared.
